# Supplementary figures and images for: Increasing transparency in machine learning through bootstrap simulation and shapely additive explanations
Source: PLoS One. 2023 Feb 23;18(2):e0281922. doi: 10.1371/journal.pone.0281922 (PMC9949629; doi:10.1371/journal.pone.0281922)

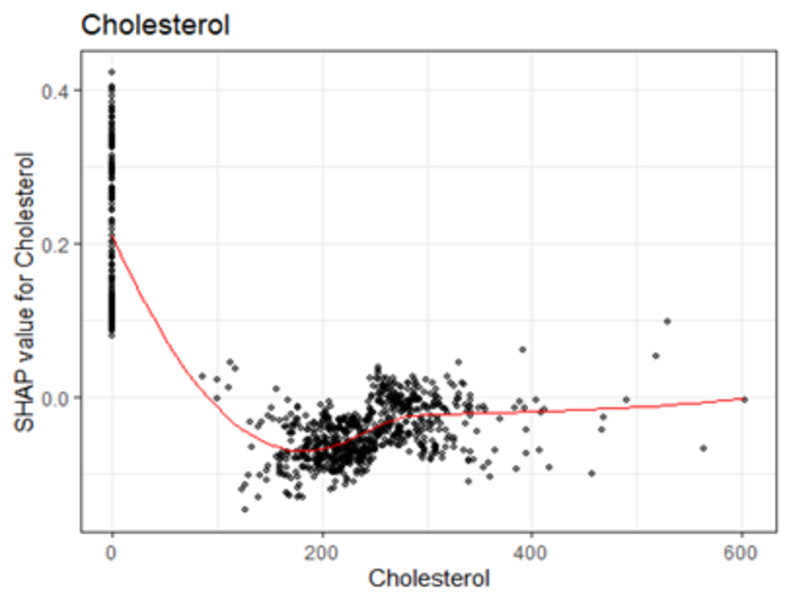

Supplement: S1 Fig — Each point represents each observation, the red line represents a trend line. X-axis is the covariate of interest, Cholesterol (mg/day). The SHAP value represents the log-odds for heart disease. (TIF) [file pone.0281922.s001.tif]

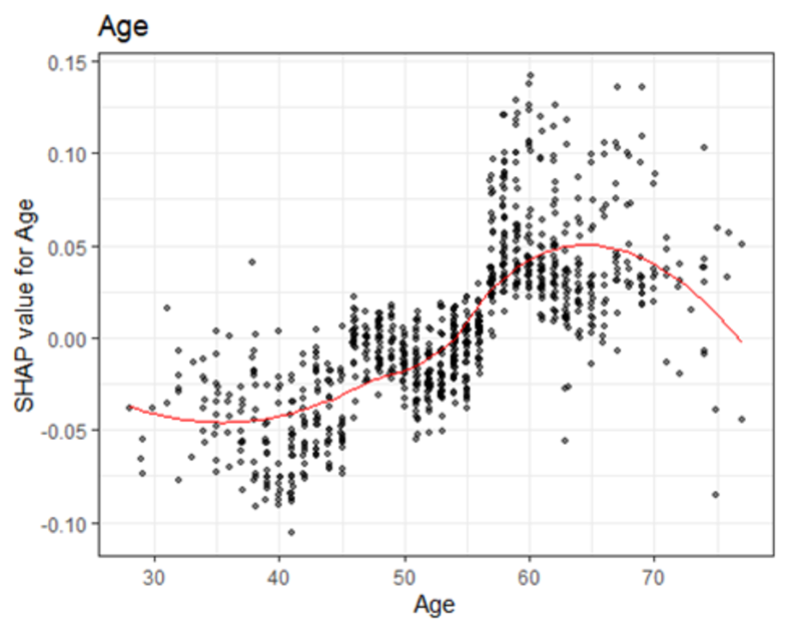

Supplement: S2 Fig — Each point represents each observation; the red line represents a trend line. X-axis is the covariate of interest, Age(years). The SHAP value represents the log-odds for heart disease. (TIF) [file pone.0281922.s002.tif]

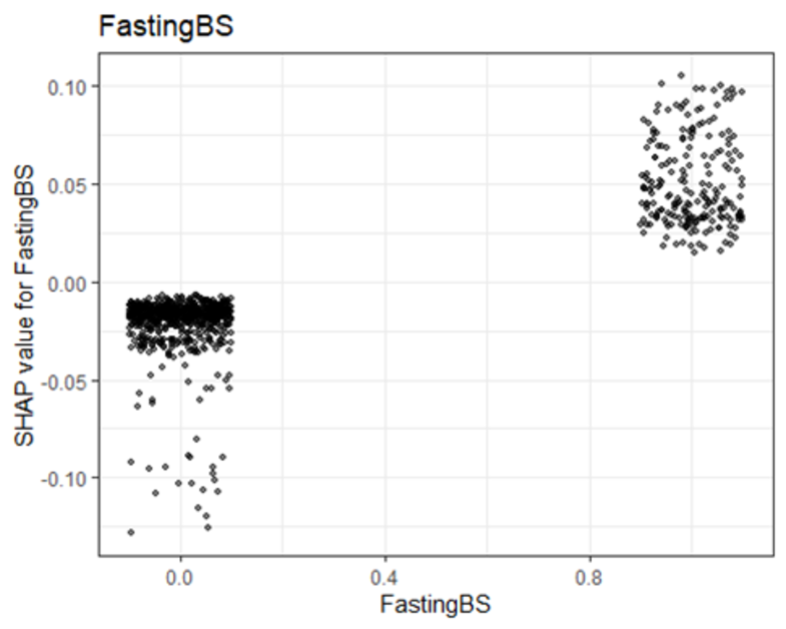

Supplement: S3 Fig — Each point represents each observation. X-axis is the covariate of interest, Fasting Blood Sugar. Non-elevated = 1, Elevated = 2. The SHAP value represents the log-odds for heart disease. (TIF) [file pone.0281922.s003.tif]

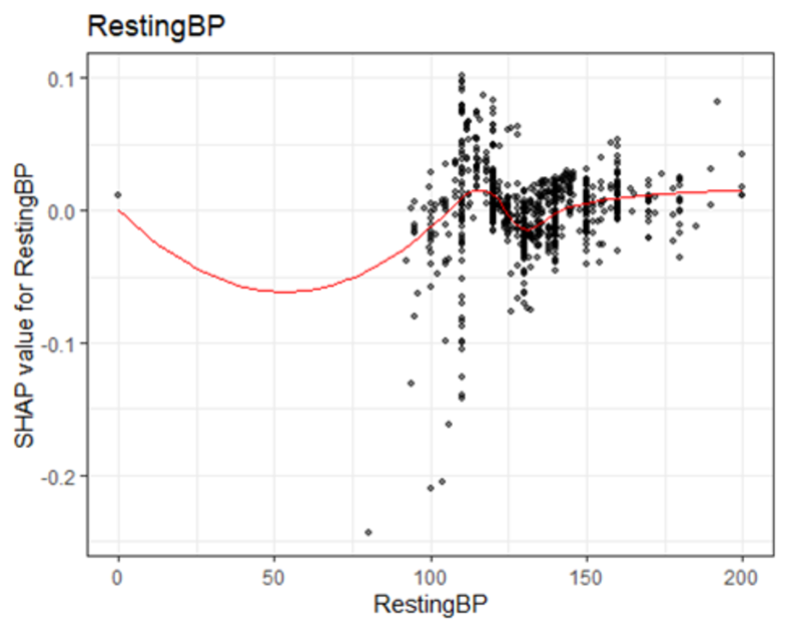

Supplement: S4 Fig — Each point represents each observation; the red line represents a trend line. X-axis is the covariate of interest, Resting Blood Pressure (mean arterial pressure). The SHAP value represents the log-odds for heart disease. (TIF) [file pone.0281922.s004.tif]

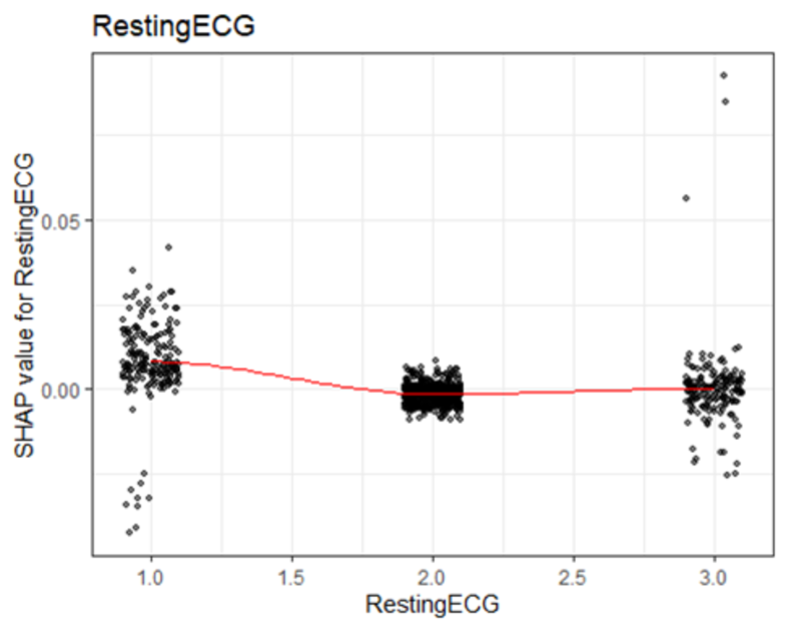

Supplement: S5 Fig — Each point represents each observation; the red line represents a trend line. X-axis is the covariate of interest, Resting Electrocardiogram. 1 represents an ST-elevation, 2 represents normal, and 3 represents left ventricular hypertrophy. The SHAP value represents the log-odds for heart disease. (TIF) [file pone.0281922.s005.tif]
