# Supplementary material for: Increasing transparency in machine learning through bootstrap simulation and shapely additive explanations
Source: PLoS One. 2023 Feb 23;18(2):e0281922. doi: 10.1371/journal.pone.0281922 (PMC9949629; doi:10.1371/journal.pone.0281922)
Supplement: Attachment — Submitted filename: SHAPLY Revisions.docx [file pone.0281922.s007.docx]

When submitting your revision, we need you to address these additional requirements.

1. **Please ensure that your manuscript meets PLOS ONE's style requirements, including those for file naming. The PLOS ONE style templates can be found at
   https://journals.plos.org/plosone/s/file?id=wjVg/PLOSOne_formatting_sample_main_body.pdf and** 
   [**https://journals.plos.org/plosone/s/file?id=ba62/PLOSOne_formatting_sample_title_authors_affiliations.pdf**](https://journals.plos.org/plosone/s/file?id=ba62/PLOSOne_formatting_sample_title_authors_affiliations.pdf)

Thank you reviewers for listing the documents leading to the correction of the style requirements.

2. Thank you for including your ethics statement: "N/A".

(1) **For studies reporting research involving human participants, PLOS ONE requires authors to confirm that this specific study was reviewed and approved by an institutional review board (ethics committee) before the study began. Please provide the specific name of the ethics committee/IRB that approved your study, or explain why you did not seek approval in this case.**

**Once you have amended this/these statement(s) in the Methods section of the manuscript, please add the same text to the “Ethics Statement” field of the submission form (via “Edit Submission”).**

**For additional information about PLOS ONE ethical requirements for human subjects research, please refer to http://journals.plos.org/plosone/s/submission-guidelines#loc-human-subjects-research.**

**(2) Please provide additional details regarding participant consent. In the ethics statement in the Methods and online submission information, please ensure that you have specified (1) whether consent was informed and (2) what type you obtained (for instance, written or verbal, and if verbal, how it was documented and witnessed). If your study included minors, state whether you obtained consent from parents or guardians. If the need for consent was waived by the ethics committee, please include this information.**

**If you are reporting a retrospective study of medical records or archived samples, please ensure that you have discussed whether all data were fully anonymized before you accessed them and/or whether the IRB or ethics committee waived the requirement for informed consent. If patients provided informed written consent to have data from their medical records used in research, please include this information**

**Once you have amended this/these statement(s) in the Methods section of the manuscript, please add the same text to the “Ethics Statement” field of the submission form (via “Edit Submission”).**

**For additional information about PLOS ONE ethical requirements for human subjects research, please refer to** [**http://journals.plos.org/plosone/s/submission-guidelines#loc-human-subjects-research**](http://journals.plos.org/plosone/s/submission-guidelines#loc-human-subjects-research)**.**

Thank you for advising on PLOS ONE requirements for ethical statements. We have updated these statements in the Methods and “ethics statement” field as well as the cover letter.

**3. Thank you for stating the following financial disclosure:**

**"The funders had no role in study design, data collection and analysis, decision to publish, or preparation of the manuscript."**

**At this time, please address the following queries:**

**a) Please clarify the sources of funding (financial or material support) for your study. List the grants or organizations that supported your study, including funding received from your institution. 
b) State what role the funders took in the study. If the funders had no role in your study, please state: “The funders had no role in study design, data collection and analysis, decision to publish, or preparation of the manuscript.”
c) If any authors received a salary from any of your funders, please state which authors and which funders.
d) If you did not receive any funding for this study, please state: “The authors received no specific funding for this work.”**

**Please include your amended statements within your cover letter; we will change the online submission form on your behalf.**

Thank you for advising on PLOS ONE’s requirements for financial disclosure. We have amended “The Authors received no specific funding for this work” to the cover letter.

4. ***In your Data Availability statement, you have not specified where the minimal data set underlying the results described in your manuscript can be found.* PLOS defines a study's minimal data set as the underlying data used to reach the conclusions drawn in the manuscript and any additional data required to replicate the reported study findings in their entirety. All PLOS journals require that the minimal data set be made fully available. For more information about our data policy, please see http://journals.plos.org/plosone/s/data-availability.**

**Upon re-submitting your revised manuscript, please upload your study’s minimal underlying data set as either Supporting Information files or to a stable, public repository and include the relevant URLs, DOIs, or accession numbers within your revised cover letter. For a list of acceptable repositories, please see http://journals.plos.org/plosone/s/data-availability#loc-recommended-repositories. Any potentially identifying patient information must be fully anonymized.**

**Important: If there are ethical or legal restrictions to sharing your data publicly, please explain these restrictions in detail. Please see our guidelines for more information on what we consider unacceptable restrictions to publicly sharing data: http://journals.plos.org/plosone/s/data-availability#loc-unacceptable-data-access-restrictions. Note that it is not acceptable for the authors to be the sole named individuals responsible for ensuring data access.**

**We will update your Data Availability statement to reflect the information you provide in your cover letter.**

Thank you for advising on PLOS ONE’s requirements for data availability. We have attached the minimal dataset and updated the cover letter.

**5. Please note that in order to use the direct billing option the corresponding author must be affiliated with the chosen institute. Please either amend your manuscript to change the affiliation or corresponding author, or email us at plosone@plos.org with a request to remove this option. Write we have new affiliation with VCU and will utilize the direct billing option.**

Thank you for advising on PLOS ONE’s affiliation with VCU. We have amended the corresponding author to utilize the direct billing option.

6. **Please upload a new copy of Figure 1 as the detail is not clear. Please follow the link for more information:
https://blogs.plos.org/plos/2019/06/looking-good-tips-for-creating-your-plos-figures-graphics/**
[**https://blogs.plos.org/plos/2019/06/looking-good-tips-for-creating-your-plos-figures-graphics/**](https://blogs.plos.org/plos/2019/06/looking-good-tips-for-creating-your-plos-figures-graphics/)

Thank you for bringing this to our attention. We have updated figures with the best resolution.

**7. Please upload a copy of Supporting Information Figures 1 to 9 which you refer to in your text on page 15.**

Thank you for bringing this to our attention, we have updated supporting figures.

8. **Please review your reference list to ensure that it is complete and correct. If you have cited papers that have been retracted, please include the rationale for doing so in the manuscript text, or remove these references and replace them with relevant current references. Any changes to the reference list should be mentioned in the rebuttal letter that accompanies your revised manuscript. If you need to cite a retracted article, indicate the article’s retracted status in the References list and also include a citation and full reference for the retraction notice.**
We have added new references to the list as advised by reviewers.

[Note: HTML markup is below. Please do not edit.]

Reviewers' comments:

Reviewer's Responses to Questions

**Comments to the Author**

1. Is the manuscript technically sound, and do the data support the conclusions?

The manuscript must describe a technically sound piece of scientific research with data that supports the conclusions. Experiments must have been conducted rigorously, with appropriate controls, replication, and sample sizes. The conclusions must be drawn appropriately based on the data presented.

Reviewer #1: Yes

Reviewer #2: Yes

2. Has the statistical analysis been performed appropriately and rigorously?

Reviewer #1: Yes

Reviewer #2: Yes

3. Have the authors made all data underlying the findings in their manuscript fully available?

The [PLOS Data policy](http://www.plosone.org/static/policies.action#sharing) requires authors to make all data underlying the findings described in their manuscript fully available without restriction, with rare exception (please refer to the Data Availability Statement in the manuscript PDF file). The data should be provided as part of the manuscript or its supporting information, or deposited to a public repository. For example, in addition to summary statistics, the data points behind means, medians and variance measures should be available. If there are restrictions on publicly sharing data—e.g. participant privacy or use of data from a third party—those must be specified.

Reviewer #1: Yes

Reviewer #2: Yes

4. Is the manuscript presented in an intelligible fashion and written in standard English?

PLOS ONE does not copyedit accepted manuscripts, so the language in submitted articles must be clear, correct, and unambiguous. Any typographical or grammatical errors should be corrected at revision, so please note any specific errors here.

Reviewer #1: Yes

Reviewer #2: Yes

5. Review Comments to the Author

Please use the space provided to explain your answers to the questions above. You may also include additional comments for the author, including concerns about dual publication, research ethics, or publication ethics. (Please upload your review as an attachment if it exceeds 20,000 characters)

Reviewer #1: This paper addresses the problem of machine learning explainability to medicine. Through bootstrap simulation and Shapley Additive exPlanations (SHAP), the study aims to increase model transparency and reliability and improve model selection.

Strengths:
1) The paper presented an interesting approach and evaluated the chosen dataset well.
2) The system presented has potential for wide-scale deployment, tested against many strong baselines.

Weaknesses:
Although the paper presented interesting contributions towards the problem of machine learning explainability in health, a few areas still need some improvement.
**• Abstract: In the abstract, only the XGBoost was mentioned as the model used, but other models, such as Random Forest, Artificial Neural Network etc was used in the study.**

Thank you for bringing this to our attention we have made the change.
• Compare **results with findings in literature: It might be worth comparing the findings** of this study with that in the literature. It is essential to know what was learnt and the methodology used.
**• SHAP over other tools: Although ML explainability is relatively new, several tools have been explored in the literature. It might be worth asking why the choice of SHAP over others, such as ELI5, LIME and Yellowbrick, etc.**

We did a literature search and found SHAP to be most prevalent over others. Additionally, SHAP has packages within R and python that are compatible with machine learning methods used in this study. Further research is needed to compare the efficacy of models such as SHAP with ELI5, LIME, and Yellowbrick, etc. Goal of paper is to generate a workflow for machine learning, taking into account bootstrapping to generate the distribution for model accuracy statistics, evaluate the feature importances of all our covariates, and highlight the utility of model explanation, only SHAP was used in the study, but we agree with the suggestions above and will do it as a line of inquiry in our next.

• Sketchy literature: For an emerging study, the literature appears insufficient missing prior work in this area. How is this study different from the below (cite if relevant):

o Dave, D., Naik, H., Singhal, S. and Patel, P., 2020. Explainable ai meets healthcare: A study on heart disease dataset. arXiv preprint arXiv:2011.03195.
o Shi, H., Yang, D., Tang, K., Hu, C., Li, L., Zhang, L., Gong, T. and Cui, Y., 2022. Explainable machine learning model for predicting the occurrence of postoperative malnutrition in children with congenital heart disease. Clinical Nutrition, 41(1), pp.202-210.
o Lu, S., Chen, R., Wei, W., Belovsky, M. and Lu, X., 2021. Understanding Heart Failure Patients EHR Clinical Features via SHAP Interpretation of Tree-Based Machine Learning Model Predictions. In AMIA Annual Symposium Proceedings (Vol. 2021, p. 813). American Medical Informatics Association.
o Zhou, Y., Chen, S., Rao, Z., Yang, D., Liu, X., Dong, N. and Li, F., 2021. Prediction of 1-year mortality after heart transplantation using machine learning approaches: A single-center study from China. International Journal of Cardiology, 339, pp.21-27.
o Chaves, J.M.Z., Chaudhari, A.S., Wentland, A.L., Desai, A.D., Banerjee, I., Boutin, R.D., Maron, D.J., Rodriguez, F., Sandhu, A.T., Jeffrey, R.B. and Rubin, D., 2021. Opportunistic assessment of ischemic heart disease risk using abdominopelvic computed tomography and medical record data: a multimodal explainable artificial intelligence approach. medRxiv.
o Obaido, G., Ogbuokiri, B., Swart, T.G., Ayawei, N., Kasongo, S.M., Aruleba, K., Mienye, I.D., Aruleba, I., Chukwu, W., Osaye, F. and Egbelowo, O.F., 2022. An Interpretable Machine Learning Approach for Hepatitis B Diagnosis. Applied Sciences, 12(21), p.11127.

Thank you for suggesting these papers. They have been helpful in revising the introduction and discussion to add greater depth. All of these papers are references either in the introduction or the discussion.

Reviewer #2: The paper is clearly written, reproducible and technically fine, in terms of statistical analyses performed.

My concern, beyond the limitations that the authors have addressed is to **discuss the approach with more complex designs (data cohorts, variables) that the one in use**.

**The design is the following**:
**Independent Variables: Demographic covariates (age and sex). Clinical covariates (Resting blood
pressure, fasting blood sugar, cholesterol, resting electrocardiogram (ECG), presence of Angina,
and maximum heart rate).
Dependent variable: heart disease, as diagnosed by a clinician.

Especially from EHR, one expects to find a mix of information sources with data of different nature, and this aspect of multimodality complicates model selection and may require different strategies to measure performance and explanation.
This might calls for more heterogeneous data to be analyzed with methods to be compared, which is in part one of the limitations that the authors have reported.
The authors should discuss more in depth this part.**

The authors would like to thank the reviewer for highlighting such an important further analysis. The authors have addressed this in the limitations and discussed it more in depth.
